# The final implant position of a commonly used collarless straight tapered stem design (Corail^®^) does not correlate with femoral neck resection height in cement-free total hip arthroplasty: a retrospective computed tomography analysis

**DOI:** 10.1186/s10195-018-0513-z

**Published:** 2018-11-13

**Authors:** Michael Worlicek, Markus Weber, Michael Wörner, Timo Schwarz, Florian Zeman, Joachim Grifka, Tobias Renkawitz, Benjamin Craiovan

**Affiliations:** 1 Department of Trauma Surgery, University Medical Center Regensburg, University of Regensburg, Franz-Josef-Strauss-Allee 1, 93053 Regensburg, Germany; 2 Department of Orthopedic Surgery, Asklepios Medical Center, University of Regensburg, Bad Abbach, Germany; 3 Center of Clinical Studies, University of Regensburg, Regensburg, Germany

**Keywords:** Hip arthroplasty, Stem version, Combined anteversion, Final stem position

## Abstract

**Background:**

In total hip arthroplasty, inadequate femoral component positioning can be associated with instability, impingement and component wear and subsequently with patient dissatisfaction. In this study, we investigated the influence of femoral neck resection height on the final three-dimensional position of a collarless straight tapered stem (Corail^®^). We asked two questions—(1) is neck resection height correlated with version, tilt, and varus/valgus alignment of the femoral component, and (2) dependent on the resection height of the femoral neck, which area of the stem comes into contact with the femoral cortical bone?

**Materials and methods:**

Three-dimensional computed tomography scans of 40 patients who underwent minimally invasive, cementless total hip arthroplasty were analyzed retrospectively. We analyzed the relationship between femoral neck resection height and three-dimensional alignment of the femoral implant, as well as the contact points of the implant with the femoral cortical bone. This investigation was approved by the local Ethics Commission (No.10-121-0263) and is a secondary analysis of a larger project (DRKS00000739, German Clinical Trials Register May-02-2011).

**Results:**

Mean femoral neck resection height was 10.4 mm (± 4.8) (range 0–20.1 mm). Mean stem version was 8.7° (± 7.4) (range − 2° to 27.9°). Most patients had a varus alignment of the implant. The mean varus/valgus alignment was 1.5° (± 1.8). All 40 patients (100%) had anterior tilt of the implant with a mean tilt of 2.2° (± 1.6). Femoral neck resection height did not correlate with stem version, varus/valgus alignment, or tilt. Independent from femoral neck resection height, in most patients the implant had contact with the ventral and ventromedial cortical bone in the upper third (77.5%) and the middle third (52.5%). In the lower third, the majority of the implants had contact with the lateral and dorsolateral cortical bone (92.5%).

**Conclusion:**

Femoral neck resection height ranging between 0 and 20.1 mm does not correlate with the final position of a collarless straight tapered stem design (Corail^®^).

**Level of evidence:**

Level 3.

## Introduction

Primary total hip arthroplasty (THA) is one of the most common and successful orthopedic operations worldwide [1]. However, malpositioning of the components is associated with an increased risk of complications, such as impingement, dislocation, pelvic osteolysis and wear, and thus early revision [2, 3]. The final position of the cup may be assessed in several ways. To date, most orthopedic surgeons rely on intraoperatively visible or palpable anatomic landmarks and aim at positioning the cup with the naked eye [4] or, alternatively, with intraoperative alignment guides [3] or recently developed computer-assisted methods [5].

According to the concept of combined anteversion, several authors have suggested first preparing the femur (‘femur first’) and then adjusting the position of the cup in accordance with femoral rotation.

The crucial point in cement-free THA seems to be positioning the femoral component. When using a common straight tapered implant, surgeons have little control about its final position. The stem follows the flexion and twist of the proximal femoral channel, ending in the ‘best-fitting’ position [6]. This final position of the stem can be viewed in three different planes. The first plane is stem version. Different studies have reported high variations in postoperative cement-free stem anteversion ranging between − 19° retroversion and up to 52° anteversion [7, 8]. The second aspect is varus/valgus alignment in the coronal plane, and the third aspect is the tilt of the component in the sagittal plane. A recent study showed that there is no correlation between native femoral version and the version of the stem implant after cement-free THA. It also showed that there seems to be no correlation between the resection height of the femoral neck and the final version of the stem implant [9]. Based on these results, we now investigated the influence of the resection height of the femoral neck on the final three-dimensional (3D) position of the femoral component in cement-free THA. To our knowledge, no study has yet analyzed the final 3D position of the femoral component and its association with the resection height of the femoral neck. In the present study, we asked two questions—(1) is neck resection height correlated with version, tilt, and varus/valgus alignment of the femoral component, and (2) dependent on the resection height of the femoral neck, which area of the stem comes into contact with the femoral cortical bone?

## Materials and methods

This study is a retrospective analysis of data obtained in a registered, prospective controlled trial (DRKS00000739, German Clinical Trials Register). The primary outcome of this larger study was to assess whether the range of motion of the prosthetic joint could be improved by computer-assisted functional optimization of position and containment of the acetabular component. The inclusion criteria were age between 50 and 75 years, an American Society of Anaesthesiologists (ASA) score ≤ 3, unilateral osteoarthritis of the hip (up to Kellgren 2 of the contralateral side), no prior hip surgery, and no hip dysplasia or trauma. Postoperative 3D computer tomography (CD) scans including the femoral condyles as well as pelvic radiographs were available for the study group.

All operations were carried out in the lateral decubitus position using the minimally invasive modified Smith-Petersen approach [10]. Press-fit cups (Pinnacle; DePuy, Warsaw, IN, USA) and cement-free hydroxyapatite-coated stems (Corail^®^; DePuy) were used. Preoperative planning was performed by using a common planning program for endoprosthesis (mediCAD; Hectec GmbH, Altdorf, Germany). Stem size was confirmed by intra- and postoperative X-rays. No obvious undersizing was detected. The Corail^®^ stem is a straight tapered cement-free stem filling the metaphysis and proximal diaphysis in the mediolateral plane. Although the position of the femoral component is dictated in part by the native femoral neck anteversion, the final position of the ‘best-fit’ stem is a compromise of fitting the straight stem down the canal of the femur and addressing the flexion and twist of the proximal femur. The extent to which anteversion of the final implant may be influenced by the surgeon [6, 7] is yet unclear. Tribological pairing consisted of polyethylene liners and metal heads with a diameter of 32 mm. Six weeks after surgery, a CT scan was obtained from the pelvis down to the femoral condyles (Somatom Sensation 16; Siemens, Erlangen, Germany). This investigation was approved by the local Ethics Committee (No. 10-121-0263). All procedures were in accordance with the ethical standards of the responsible committee on human experimentation and with the Helsinki Declaration of 1975, as revised in 2000.

**Fig. 1:**
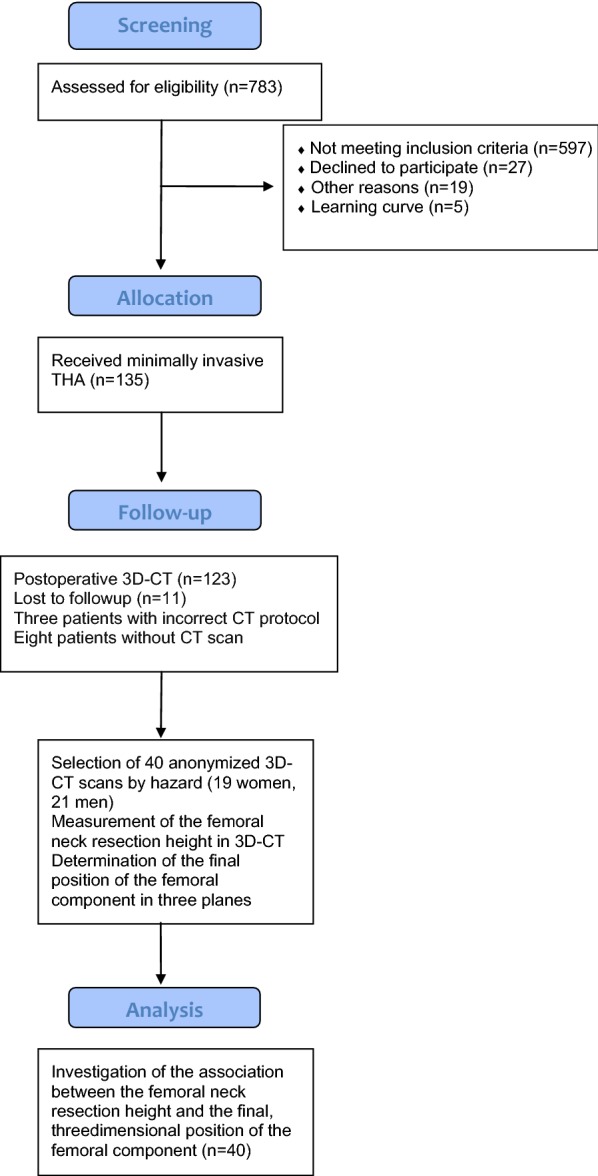
Consolidated Standards for Reporting Trials flow diagram for participants. (*THA* total hip arthroplasty)

**Fig. 2:**
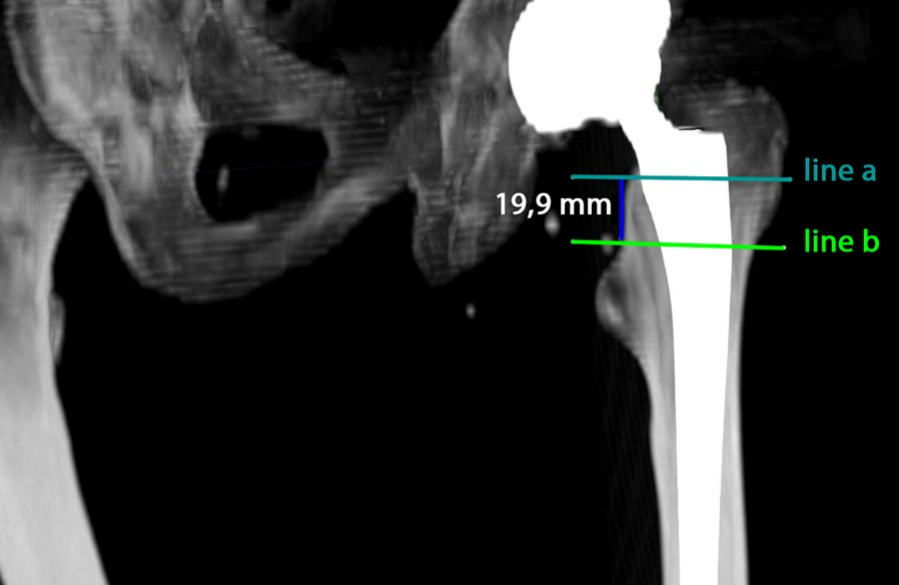
Measurement of femoral neck resection height. Line **a** represents the deepest point of the resection line and line **b** the proximal basis of the lesser trochanter

**Fig. 3:**
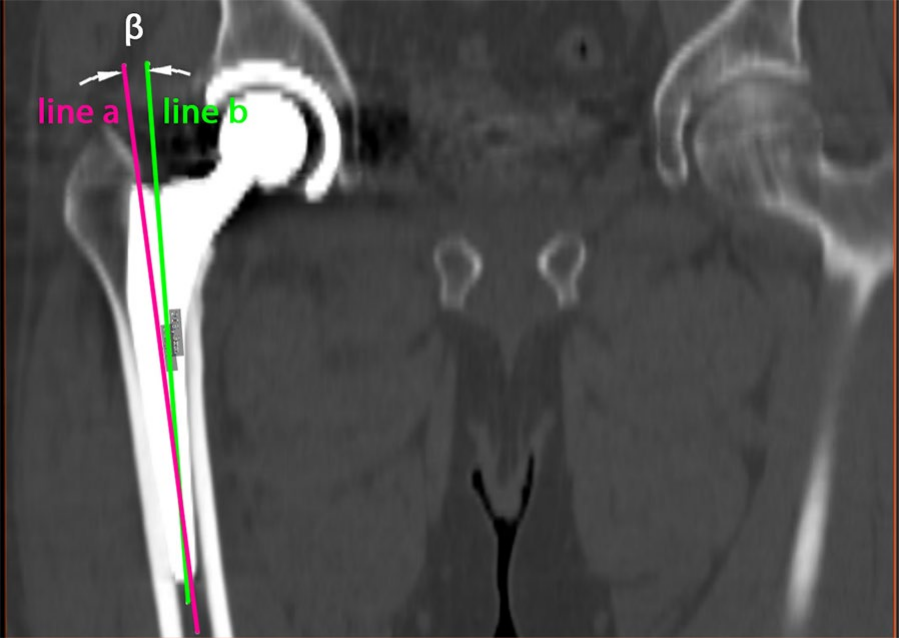
Measurement of the alignment of the implant in relation to the femoral shaft axis in the coronal plane (varus/valgus). Line **a** represents the femoral shaft axis, line **b** the implant shaft axis. In this case the alignment angle β was 5.7° varus

**Fig. 4:**
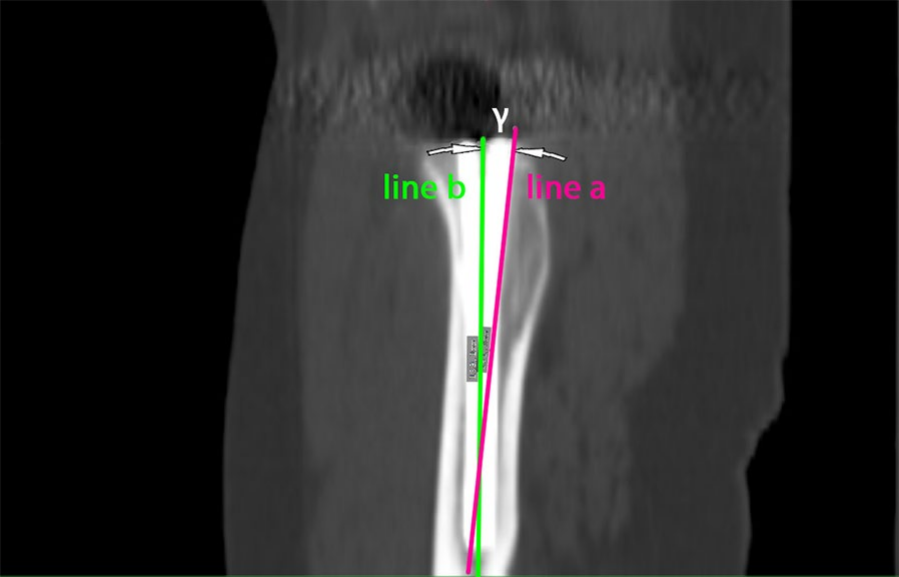
Measurement of the alignment of the implant in relation to the femoral shaft axis in the sagital plane (tilt). Line **a** represents the femoral shaft axis, line **b** the implant shaft axis. In this case the alignment angle γ was 2.6° anterior tilt

For the current study, due to the complex measurement protocol, 3D CT scans of 40 patients (19 women, 21 men) were chosen randomly from the anonymized whole study collective by an independent observer, analyzed and finally deanonymized (Fig. 1). Characteristics of the study group are shown in Table 1. Femoral neck resection height was defined as the distance between the deepest point of the resection line and the proximal basis of the lesser trochanter (Fig. 2). Resection height and the angle of the femoral component relating to the femoral axis in the sagittal and the coronal plane were measured with the ‘semi-automatical’ function of a newly developed digital planning software for CT scans (Modicas, Erlangen, Germany) (Figs. 3, 4). This software offers the possibility to assess hips in three dimensions, to exactly determine the axes, and to automatically calculate angles. The alignment of the implant in relation to the femoral shaft axis in the coronal plane was defined as varus/valgus deviation (Fig. 3).

**Table 1:** Study group characteristics

	*n* = 40
Sex (female) (%)	19 (47.5)
Age (years)	61.1 (SD 7.1)
BMI (kg/m^2^)	26.8 (SD 4.3)
ASA	1.9 (SD 0.7)
Treatment side (right) (%)	19 (47.5)

For categorical data, values are given as relative and absolute frequencies; for quantitative data, values are given as mean with SD (standard deviation) in parentheses

*BMI* body mass index, *ASA* American Society of Anaesthesiologists

The alignment of the implant in relation to the femoral shaft axis in the sagital plane was defined as tilt (Fig. 4).

**Fig. 5:**
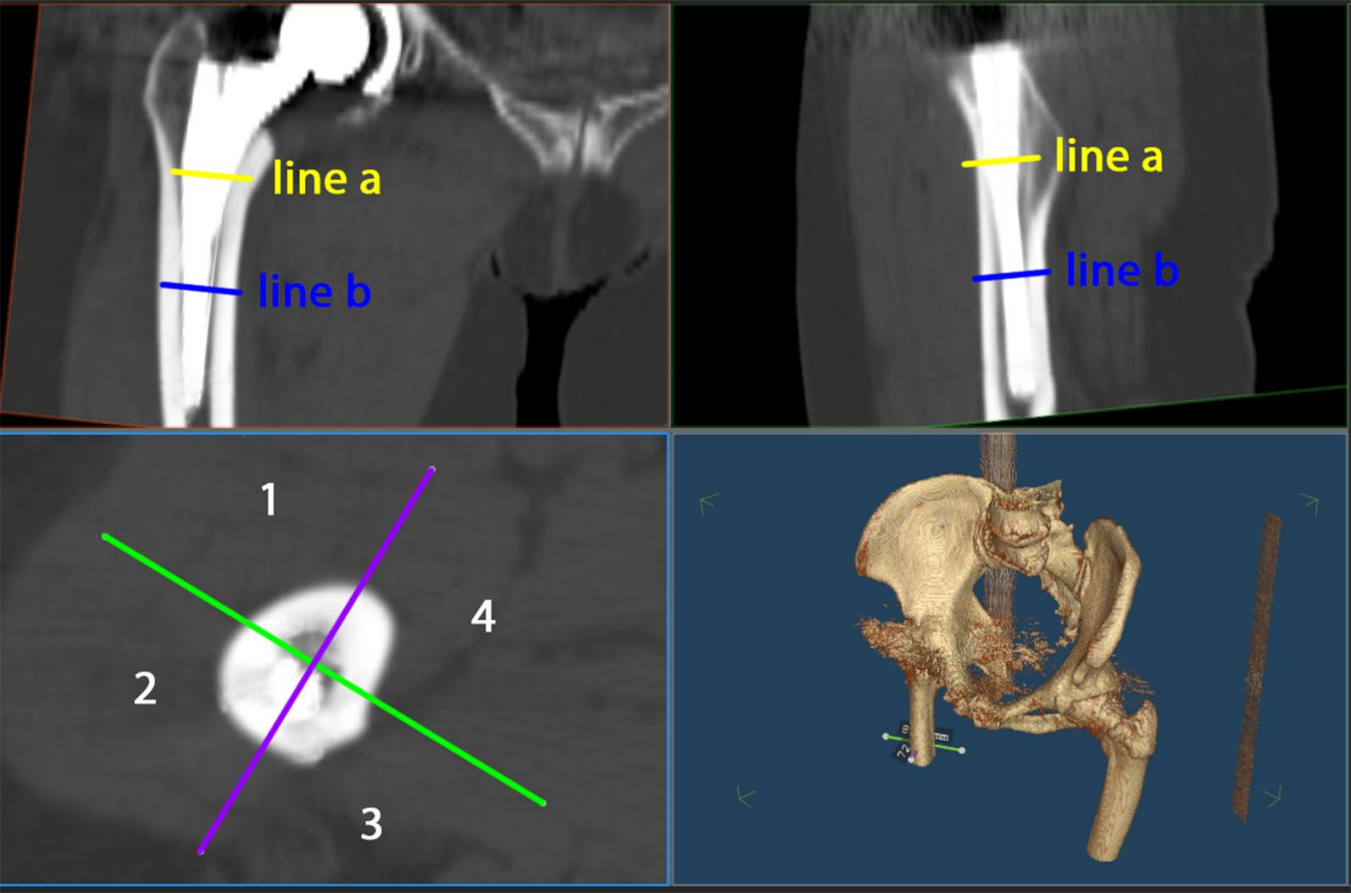
The screenshot of the used software shows the contact points of the implant with the femoral cortical bone. In this example, the implant has contact with the ventral cortical bone in the upper and middle third and with the dorsolateral cortical bone in the lower third. 1 = ventral, 2 = lateral, 3 = dorsal, 4 = medial

In addition, 3D CT assessment of the prosthetic stem version was obtained by an independent, blinded external institute (MeVis Medical Solutions, Bremen, Germany) as described by Sendtner et al. [7]. For assessing the contact points between implant and femoral cortical bone, the stem was virtually subdivided into three parts—the proximal, middle, and distal third. In the axial, coronal, and sagittal plane, we analyzed which third of the stem came into contact with the anterior, posterior, medial, or lateral part of the femoral cortical bone (Fig. 5).

All radiological measurements were carried out by one of the authors (MW) who was familiar with the software.

### Statistical methods

The influence of height and alignment of the implant on femoral neck resection was analyzed using simple linear regression models. Differences in neck resection height regarding the position of the implant in relation to the femoral cortical bone were analyzed with analyses of variance (ANOVA). A *p* value of < 0.05 was considered statistically significant. All analyses were carried out with *R 3.3.1* (R Foundation for Statistical Computing, Vienna, Austria).

## Results

### Femoral neck resection height and alignment of the implant

**Fig. 6:**
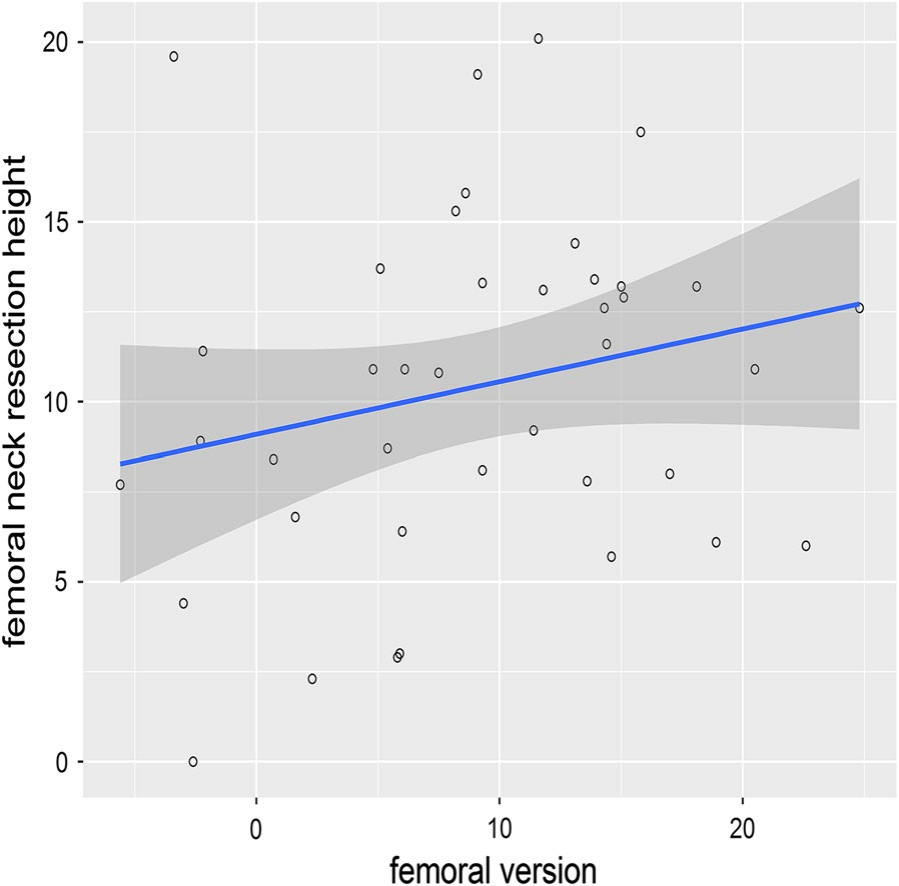
The scatterplot shows the correlation between femoral version and femoral neck resection height. The blue line indicates the linear regression line and the dark grey area the corresponding the 95% CI

Mean neck resection height was 10.4 mm (± 4.8), ranging from 0 to 20.1 mm. Mean stem version was 8.7° (± 7.4), ranging from − 2° to 27.9° (Fig. 6).

**Fig. 7:**
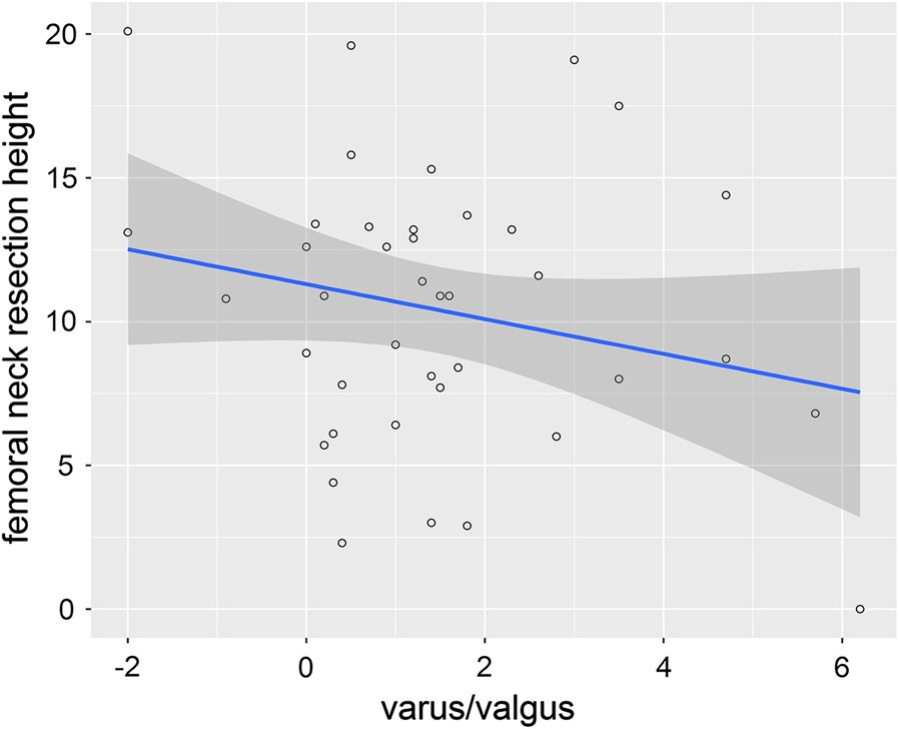
The scatterplot shows the correlation between varus/valgus alignment and femoral neck resection height. The blue line indicates the linear regression line and the dark grey area the corresponding the 95% CI

**Fig. 8:**
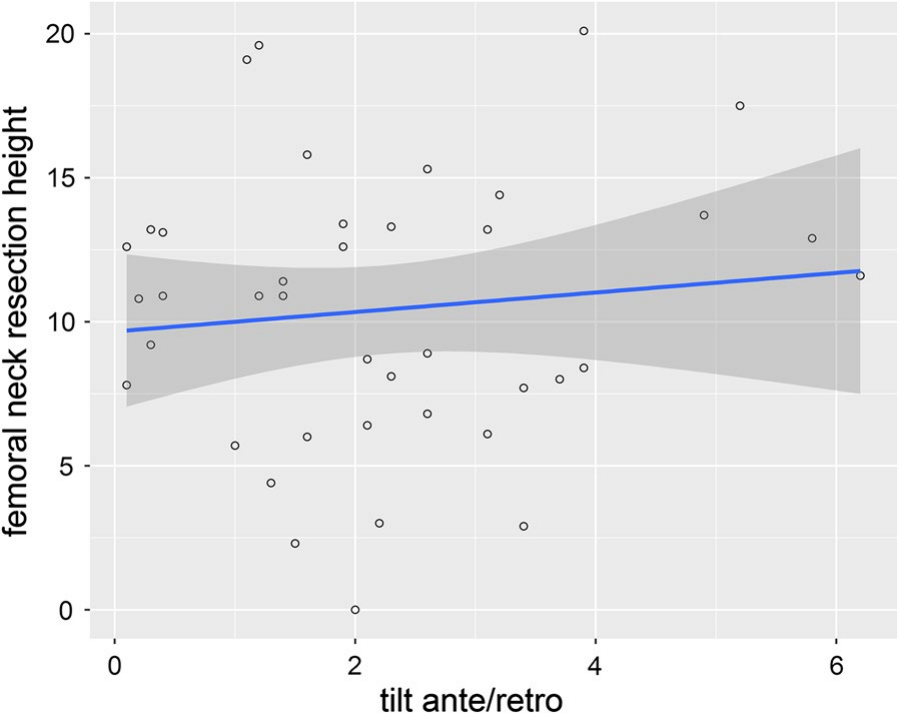
The scatterplot shows the correlation between implant tilt and femoral neck resection height. The blue line indicates the linear regression line and the dark grey area the corresponding the 95% CI

Thirty-seven patients (92.5%) had varus alignment of the implant, while only three patients (7.5%) had valgus alignment. The mean varus/valgus alignment was 1.5° (± 1.8); positive values represented varus alignment, negative values valgus alignment (Fig. 7). All 40 patients (100%) showed anterior tilt of the implant with a mean tilt of 2.2° (± 1.6) (Fig. 8).

Femoral neck resection height was not correlated with stem version, varus/valgus alignment, or tilt. Slope (95% coefficient interval; CI), coefficient of determination, and *p* values of the linear regression models are shown in Table 2.

**Table 2:** 95% coefficient interval (CI), coefficient of determination (*R*^2^), *p* value of femoral neck resection in relation to implant alignment

	B (95%-CI)	*R* ^2^	*p* value
Varus/valgus	− 0.61 (− 1.47, 0.25)	0.051	0.161
Tilt	0.34 (− 0.67, 1.34)	0.012	0.499
Femoral version	0.15 (− 0.05, 0.35)	0.054	0.148

Femoral neck resection height and position of the implant in relation to the femoral cortical bone

### Upper third of the implant

In 20 patients (50%), the implant came into contact with the ventral cortical bone, in 11 patients (27.5%) with the ventromedial cortical bone, in four patients (10%) with the medial cortical bone, and in one patient (2.5%) with the dorsal, ventrolateral, or dorsomedial cortical bone. Two patients did not show any contact with the upper third of the implant. No significant differences (ANOVA, *p* = 0.179) were found when comparing the resection height of patients with contact with the ventral cortical bone (11.4 ± 4.8) to that of patients with contact to the ventromedial cortical bone (7.9 ± 5.0) and medial cortical bone (10.0 ± 5.0).

### Middle third of the implant

In 16 patients (40%), the implant came into contact with the ventral cortical bone, in five patients (12.5%) with the ventromedial cortical bone, in four patients (10%) with the ventrolateral cortical bone, in three patients (7.5%) with the medial cortical bone, in six patients (15%) with the lateral cortical bone, and in one patient (2.5%) with the medial and lateral cortical bone. Five patients did not show any contact with the middle third of the implant. No significant difference in resection height was found between the contact areas (ANOVA, *p* = 0.449).

### Lower third of the implant

In 28 patients (70%), the implant came into contact with the dorsolateral cortical bone, in nine patients (22.5%) with the lateral cortical bone, in two patients (5%) with the ventral cortical bone, and in one patient (2.5%) with the dorsal, lateral, and medial cortical bone. No difference in resection height was found between the contact areas in the lower third of the implant (ANOVA, *p* = 0.862).

## Discussion

In answer to the first question posed by this study, we showed that the final position of the stem we used in this clinical trial is not related with the resection height of the femoral neck. We did not find any clinically relevant correlation between neck resection height and version, tilt, or varus/valgus alignment of this commonly used cement-free, hydroxyapatite-coated, straight tapered femoral stem. We therefore concluded that orthopedic surgeons have only little control about the final position of the stem when using this special straight tapered implant in THA. Subsequently, intraoperative measurement of the femoral stem version is crucial for surgeons aiming for optimized combined anteversion of the cup and stem.

This fact leads directly to our second question. We did not find any correlation between femoral neck resection height and the area of the stem we used in our study coming into contact with the femoral cortical bone. Independent of neck resection height, the majority of implants come into contact with the metaphyseal area (upper third) of the ventral and/or medial cortical bone. Furthermore, most implants come into contact with the middle and distal third of the dorsolateral or only the lateral cortical bone.

Our study has several limitations. First, we used the single cement-free stem from only one manufacturer. The Corail^®^ stem is a clinically successful implant made of forged titanium alloy (TiAl6V4) [11, 12]. The implant is straight with a quadrangular cross-section. The proximal part is flared in the sagittal and coronal plane to provide 3D stabilization in the metaphyseal area. Therefore, our findings may not be transferable to other stem designs, such as wedge-hip-stems that provide stabilization in the diaphyseal area. Second, we used a minimally invasive anterolateral approach with the patient in the lateral decubitus position. Theoretically, the surgical approach (anterior, antero-lateral, lateral, or dorsal) may have an impact on final stem anteversion, as shown by Bernasek et al. who found a higher prevalence of varus stem outlier in a minimally invasive modified Watson-Jones approach compared to a lateral approach [13].

Third, the mean resection height in our study collective was 10.4 mm (± 4.8), which was the goal of our surgical team. Higher or deeper osteotomy may cause deviations in femoral component rotation and should be considered in subsequent studies. Fourth, the individual anatomy of the femoral neck, shaft and medullary canal can vary, so femoral neck resection height sometimes needs to be adapted.

A wide range of stem versions for cement-free THA have been described in the literature. Sendtner et al. found cement-free stems ranging between − 19° retroversion and up to 33° anteversion. These findings were in accordance with the results of Wines et al. and Bargar et al. who described a postoperative range of cement-free stem versions from − 15° up to 52° and 1° up to 39°, respectively [7, 8]. These ranges are mainly caused by the natural anteroposterior and mediolateral bow of the femoral canal, the thickness of the posterior cortex, and the width of the medullary canal [14–16]. Our study confirmed this wide range of rotation in cement-free stems ranging from − 2.0° retroversion to 27.9° anteversion. Our results are in contrast to the findings of Dimitrou et al. who showed a correlation between the osteotomy angle and femoral version and between the level of the osteotomy and the frontal plane of the stem, respectively. They also used a non-anatomical straight tapered stem, but a posterolateral approach. Therefore, this approach might have influenced their results, as mentioned before.

To our knowledge, no study has so far considered the influence of femoral neck resection height on the final position and alignment of a straight tapered cement-free implant. We therefore believe that our trial contributes to the understanding of the concept of cement-free THA for this stem design.

In conclusion, we showed that femoral neck resection height ranging between 0 and 20.1 mm does not correlate with the final position of a straight tapered cement-free implant. Thus, surgeons cannot control the final position of this specific implant by adapting femoral neck resection height. For exact modulation between cup and stem we recommend the concept of combined anteversion/‘femur first’ and the use of an image-free navigation system.
